# Electrochemical Sensors Based on Transition Metal Materials for Phenolic Compound Detection

**DOI:** 10.3390/s24030756

**Published:** 2024-01-24

**Authors:** Isilda Amorim, Fátima Bento

**Affiliations:** 1 Centre of Chemistry, University of Minho, Gualtar Campus, 4710-057 Braga, Portugal; 2 Clean Energy Cluster, International Iberian Nanotechnology Laboratory (INL), Avenida Mestre Jose Veiga, 4715-330 Braga, Portugal

**Keywords:** electrocatalysts, electrochemical sensors, phenolic compounds, transition metal oxides, transition metal chalcogenides, transition metal phosphides

## Abstract

Electrochemical sensors have been recognized as crucial tools for monitoring comprehensive chemical information, especially in the detection of a significant class of molecules known as phenolic compounds. These compounds can be present in water as hazardous analytes and trace contaminants, as well as in living organisms where they regulate their metabolism. The sensitive detection of phenolic compounds requires highly efficient and cost-effective electrocatalysts to enable the development of high-performance sensors. Therefore, this review focuses on the development of advanced materials with excellent catalytic activity as alternative electrocatalysts to conventional ones, with a specific emphasis on transition metal-based electrocatalysts for the detection of phenolic compounds. This research is particularly relevant in diverse sectors such as water quality, food safety, and healthcare.

## 1. Introduction

Sensors are rapidly emerging as advanced technologies with a wide range of applications, offering unique opportunities to obtain comprehensive chemical information. They play a crucial role in detecting and monitoring hazardous or harmful analyte traces. These devices are designed for in situ or ex situ assessments of samples, making them invaluable for health and environmental monitoring and food quality control, ultimately enhancing the overall quality of modern life [1,2].

Over the years, traditional analytical methods such as high-performance liquid chromatography (HPLC), gas chromatography-mass spectrometry (GC-MS), UV/Vis spectroscopy, X-ray fluorescence spectroscopy (XFS), capillary electrophoresis (CE), atomic absorption spectroscopy (AAS), inductively coupled plasma mass spectroscopy (ICP-MS), and inductively coupled plasma optical emission spectroscopy (ICP-OES) have been established for the detection and quantification of different analytes at low levels. However, these analytical techniques have limitations that include sample preparation, clean-up and pre-concentration processes, the need for sophisticated instrumentation, and an expert operator. Furthermore, they can be expensive, tedious, and time-consuming [1,3]. To overcome these limitations, researchers have devoted significant efforts to developing efficient methods and devices for the rapid and sensitive analysis of different analytes. To this end, the development of sensing devices is of great interest as they offer unique characteristics such as miniaturization, low cost, ease-of-use, specificity, selectivity, and real-time monitoring capabilities [1].

A sensor’s performance is usually evaluated based on its limit of detection (LOD), linear ranges, sensitivity, reproducibility of response, selectivity, and its response to interferences. Other parameters include the sensor’s response time, operational and storage stability, and a regenerable sensing surface for several consecutive measurements [4]. 

Significant progress in the improvement of detection performance for monitoring drugs, organic and inorganic pollutants, and other various molecules has been made in the last few years. Recently, nanomaterial-based sensors have shown great potential in improving the detection of several analytes due to their high surface reactivity, large surface area, strong adsorption capacity, and high catalytic efficiency [3]. Therefore, the design of advanced materials with good catalytic activity to detect molecules is key to constructing a sensitive sensor. For instance, transition metal-based materials such as oxides (TMOs), phosphides (TMPs), and chalcogenides (TM-S, Se, and Te) represent an important class of compounds with good electrical conductivity, durability, and high catalytic activity, with some of them possessing metalloid characteristics [5,6,7]. They have gained a widespread reputation as promising electrode materials in the field of energy conversion and storage [6,7]. Meanwhile, they have gradually received increasing consideration for their new feature as electrode material in electrochemical sensing for different applications. 

Phenolic compounds are molecules that contain at least one hydroxyl group directly bonded to one or more aromatic rings [8]. Phenolic compounds can occur naturally in the environment, acting as flavors and pigments in many plant foods (fruits, vegetables, cereals, beans, etc.) and beverages (tea, coffee, beer, wine, etc.) and have shown interesting bioactivities, such as antioxidant, antimicrobial, and anti-inflammatory activities [9]. Also, they can originate from anthropogenic sources such as paper manufacturing, agriculture, pharmaceuticals, dyes, and pesticide production or from the petrochemical industry [8,10]. Many phenolic compounds are among the chemicals of major concern and have been designated as priority pollutants by the US Environmental Protection Agency (US EPA) and European Commission (EC) [11,12], particularly chlorophenols and nitrophenols due to their toxicity and persistence in the environment, accumulating and exerting toxic effects on living systems including humans [13,14]. 

Bioactive molecules containing phenolic moieties, such as dopamine, ascorbic acid, and uric acid, are of major biomedical concern, playing significant roles in human metabolism. Their imbalances in the human body may indicate several serious diseases (e.g., Parkinson’s, schizophrenia, hyperuricemia, etc.) [15,16]. 

Thus, the design of advanced materials that have good catalytic activity for phenolic compound detection and are simultaneously accessible is relevant for the efficient control of diverse sectors like water quality, food safety, and healthcare. The mass production of affordable sensors for phenolic compound monitoring is required for both environmental protection and public health. 

This review intends to provide a snapshot of the current development of nanostructured electrode materials based on transition metal materials for electrochemical sensing of phenolic compounds. Over the past seven years, there has been a significant increase in the number of publications addressing the sensing of phenolic compounds and the utilization of transition metal materials for sensor development. This heightened research attention highlights the growing interest in phenolic sensing and the application of transition metals in this context. A comprehensive search conducted on ISI Web of Science revealed noteworthy trends. When employing the keywords “sensor” and “phenolic compounds”, we observed a doubling of publications in the last seven years (2018–2023) compared to the preceding period of 2010–2017. Interestingly, during the 2018–2023 timeframe, there were 632 publications; only 73 identified as reviews in the earlier period. Similarly, utilizing the keywords “transition metal materials” and “sensor”, we observed a nearly fourfold increase in the volume of publications during the 2018–2023 timeframe compared to 2010–2017. An impressive total of 3521 publications were identified, with 647 reviews specifically focused on this area. These data underscore the substantial growth in research attention toward phenolic sensing and in the application of transition metals in sensing over the past seven years. Importantly, as far as we know, there is currently no existing review that comprehensively combines these areas of interest. Therefore, this review holds significant importance for readers interested in exploring emerging strategies and research trends in transition metal-based sensors developed for the detection of phenolic molecules. Our work aims to bridge this gap by providing a thorough and focused analysis of the latest advancements in this specific research area, offering readers a valuable resource in the rapidly evolving landscape of sensor research.

## 2. Transition Metal-Based Electrocatalysts

Catalysts are essential materials for a modern and sustainable society, as industrial activities heavily rely on catalytic processes or materials. The demand for the production of low-cost, efficient electrocatalytic materials is critical for the effective deployment of current technologies. 

In the past, mercury was established as the electrode material of choice for analytical electrochemistry due to its ability to form reproducible surfaces easily [17]. However, due to some drawbacks, such as disposal issues, contamination, poisoning, and toxicity, the use of mercury as an electrode material has become severely limited and obsolete [18]. In the pursuit of more environmentally friendly electrode materials, nanostructured catalytic materials based on noble metal catalysts (e.g., Ag, Au, Pt, Pd, Ir, and their alloys) are widely acknowledged as the most efficient and are extensively used in various electrochemical applications [19,20,21] including energy conversion and storage, environmental remediation, and chemical production in both laboratories and industrial sectors. However, noble metals are scarce, consequently limiting their large-scale application. Therefore, the replacement of noble metals with abundant, cost-effective, and accessible alternatives with high activity and stability is highly desirable, shifting researcher’s interest toward developing transition metal-based electrocatalysts, including pure metals, alloys, metal oxides, sulfides, selenides, phosphides, carbides, nitrides and their composites [22,23,24]. 

Electrocatalysts based on 3d transition metals (TMs), such as Mn, Fe, Co, and Ni, represent a typical class of non-precious metal-based materials. TM-based catalysts exhibit facile redox properties, good conductivities, and high chemical stability and demonstrate good catalytic performance in different applications, such as supercapacitors, batteries, and sensors [25].

Indeed, catalysts based on transition metals have demonstrated remarkable capabilities in the sensitive detection of various significant molecules, such as the enzymeless electrochemical detection of glucose and hydrogen peroxide (H_2_O_2_). For instance, a range of monometallic and bimetallic TMs, including Co_3_O_4_, NiSe_2_, NiS, Ni_2_P, Co_2_P, NiCo_2_O_4_, NiCoP, and CoNi_2_Se_4_ have demonstrated promising results in the oxidation of glucose and H_2_O_2_ [24,26,27,28]. Moreover, TM-based materials have also been reported for efficient electrochemical sensing of other small inorganic molecules, such as phosphates [29,30], nitrites [31,32,33], sulfadiazine [34,35], and superoxide anions [36]. This broad spectrum of applications highlights the versatility of TMs in electrochemical sensing.

Beyond the inherent advantages such as cost-effectiveness, stability, reproducibility, and simplicity in development, the mechanism underlying the use of TMs in electrochemical sensing relies on the redox couple of the multivalent metal. This process involves the construction of medium-strength bonds with substrates, utilizing the unpaired d-electrons from the d-orbital for mediating electron transfer mediation. Consequently, this enhancement in the adsorption or desorption rates of the analyte leads to a more robust current response [28,37].

However, despite their notable attributes, they have not yet surpassed the benchmark set by noble metal-based counterparts. Therefore, substantial efforts are required to enhance their efficiency and performance. Various strategies, including morphology design, composition tuning, and surface engineering, have been widely employed to improve the electrocatalyst’s performance by exposing more accessible active sites for catalytic reactions and enhancing the intrinsic reactivity of each active site [25,38].

### 2.1. Transition Metal Oxides

**Figure 1:**
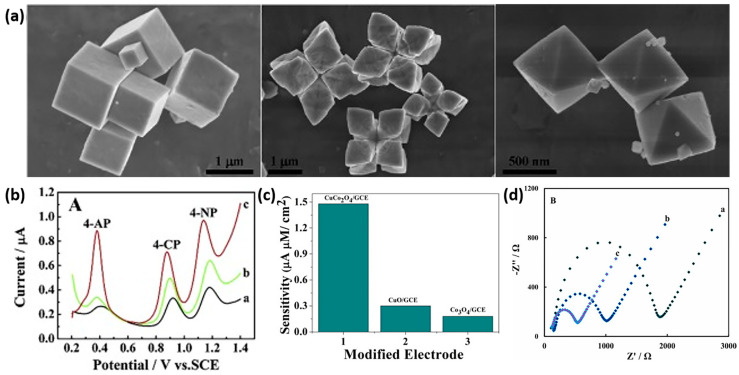
(**a**) SEM images of Cu_2_O crystals with various morphologies: cubes, stars, and octahedrons. (**b**) DPV curves of three phenols on C-Cu_2_O/GCE (black curve), S-Cu_2_O/GCE (green curve), and O-Cu_2_O/GCE (red curve). Reproduced with permission from [39]. (**c**) Comparison in the sensitivity for the oxidation of metol at different modified electrodes. Reproduced with permission from [43]. (**d**) Nyquist plot of EIS for bare GCE (black curve), CuFe_2_O_4_/GCE (blue curve), and MWCNTs/CuFe_2_O_4_/GCE (purple curve). Reproduced with permission from [47].

From the perspective of electrodes, several transition metal (TM)-based electrocatalysts, particularly oxides, have been identified as efficient materials for the detection of phenolic compounds. For instance, Gan et al. reported cubic (C)-, starlike (S)-, and octahedral (O)-shaped Cu_2_O polyhedrons (Figure 1a) for the simultaneous electrochemical sensing of 4-aminophenol (4-AP), 4-chlorophenol (4-CP), and 4-nitrophenol (4-NP), known as harmful water pollutants. Despite all morphologies displaying three independent oxidation peaks, the electrochemical activity was found to be morphology-dependent, following the order C–Cu_2_O < S–Cu_2_O < O–Cu_2_O (Figure 1b). The authors suggest that the enhanced electrocatalytic activity of the O–Cu_2_O for sensing the three phenols results from its high specific surface area and relatively rich {111} facets compared to the other morphologies. Wrapping GO nanosheets around O–Cu_2_O particles resulted in an improved signal for 4-AP, 4-CP, and low detection limits. Moreover, the proposed sensor exhibited a relative error value below 5% when compared with the HPLC technique for detecting these phenolic compounds in industrial wastewater [39]. Using a simple and low-cost hydrothermal method, puffy ball-like cobalt oxide nanostructures were synthesized for the non-enzymatic electrochemical detection of uric acid (UA). Early detection of elevated UA levels is essential to prevent potential health issues. The modified glassy carbon electrode (GCE) with the proposed material showed a sensitivity of 2.16 A M^−1^ cm^−2^. Alongside its excellent selectivity, long-term stability, and reproducibility, the sensor demonstrated practical applicability in human serum samples [40]. A mesoporous Co_3_O_4_ with an ultrathin sheet-like morphology, developed through a sacrificial template method followed by an annealing process, proved to be efficient for the simultaneous detection of hydroquinone (HQ) and catechol (CC). These two common isomers are widely used in industries such as cosmetics, dyes, pesticides, and plasticizers and are harmful to both the environment and humans. The Co_3_O_4_ modified GCE displayed a linear current response with increasing concentrations of HQ and CC in the range of 1–500 µM, achieving a limit of detection of 0.1 µM for both analytes using differential pulse voltammetry (DPV) [41].

Bimetallic oxides have also been developed for the detection of phenolic compounds. Recent studies have demonstrated that the incorporation of a secondary transition metal into a monometallic TM can significantly improve the electrochemical performance, likely due to the synergistic effect between different metal species, creating lattice dislocations and defects that increase active sites [42]. Through hydrothermal synthesis, CuCo_2_O_4_ nanorods were developed for the detection of metol (N-methyl-p-aminophenol sulfate), a chemical widely used in the photography industry that has been found to be carcinogenic and can contaminate water bodies. Compared with monometallic CuO and Co_3_O_4_ counterparts modified on the GCE, CuCo_2_O_4_/GCE exhibited a higher current response with a lower oxidation potential and, therefore, higher sensitivity (Figure 1c). A low limit of detection of 6 nM was achieved for CuCo_2_O_4_/GCE. The authors attribute the good electrochemical performance to the higher surface area, facilitated electron transfer rate, and optimum electronic properties [43]. Liu et al. prepared CoFe_2_O_4_ nanoparticles obtained through a sol–gel combustion method as an electrode material for the detection of bisphenol A (BPA), widely used in the synthesis of common plastic products that can be released into food products and water sources, acting as an endocrine-disrupting chemical. The CoFe_2_O_4_/GCE sensor exhibited a low limit of detection of 3.6 nM in the linear range of 0.05–10 µM, capable of detecting BPA in tap water and milk samples, with good recoveries [44]. 

Carbon materials such as carbon nanotubes (CNTs) and reduced graphene oxide (rGO) can be used to incorporate TMs for the construction of nanocomposites for sensing applications due to their high surface area and mechanical stability as well as to improve the conductivity of the hybrid material. For example, graphitic-carbon nitride (g-C_3_N_4_) was used to entrap zinc ferrite nanoparticles (ZnFe_2_O_4_ NPs) to construct a GCE-modified sensor for the electrochemical detection of 4-NP. The current responses of this sensor increased linearly with concentrations from 0.015 to 724.17 µM, showing a sensitivity of 1.68 A M^−1^ cm^−2^ using amperometric analysis [45]. An improved sensitivity (36.9 A M^−1^ cm^−2^) for the detection of 4-NP was obtained for a porous three-dimensional ZnFe_2_O_4_/PANI@rGO aerogel synthesized via hydrothermal reaction and freeze-drying processes. The good electrochemical sensing performance of the proposed sensor was ascribed to the synergies between the local conductivity of the PANI@rGO nanosheets and the conductivity of the three-dimensional graphene aerogel frameworks, while the interconnected frameworks may provide more active sites, which is favorable for mass transport [46]. Another nanocomposite based on MWCNTs/CuFe_2_O_4_ was successfully applied for BPA determination. The nanocomposite showed a much-improved electrochemical response toward BPA detection compared to the GCE and unsupported CuFe_2_O_4_ due to the enhanced electron transfer rate, as proven by electrochemical impedance spectroscopy (EIS) (Figure 1d) with decreased charge transfer resistance (R_ct_). Moreover, in real water samples (tap and mineral water), good recovery ranges from 95.8% to 103.1% were obtained [47]. A hybrid electrochemical sensing system designed for the detection of chlorogenic acid (CGA) was developed by Yang et al. This innovative system is based on mesoporous nitrogen-rich carbon on the surface of ultrathin-reduced graphene oxide (mNPC@rGO) combined with NiFe_2_O_4_. Density functional theory calculations revealed that the incorporation of NiFe_2_O_4_ into mNPC@rGO not only generated Fe sites with enhanced catalytic activity but also optimized the adsorption of intermediates through modulation of the electronic structure, thereby enhancing overall catalytic performance. Consequently, the sensor exhibited excellent sensing capabilities, featuring a wide linear range of 0.0001–20 μM and impressively low limits of detection at 0.02 nM. This remarkable performance renders the sensor highly promising for the effective detection of CGA in natural samples [48].

### 2.2. Transition Metal Chalcogenides

As TM oxides typically exhibit low electrical conductivity, alternative TM-based electrocatalysts such as sulfides, selenides, and tellurides have been explored for the electrochemical sensing of phenolic compounds.

#### 2.2.1. Transition Metal Sulfides

**Figure 2:**
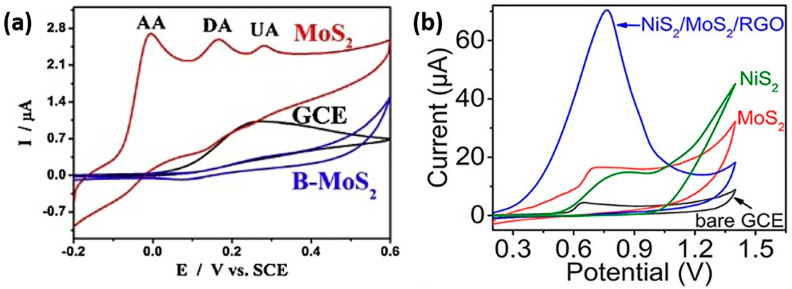
(**a**) CV curves of GCE, bulk-MoS_2_/GCE, and MoS2/GCE with all biomolecules. Reproduced with permission from [49]. (**b**) CV curves of bare GCE, NiS_2_, MoS_2_, and NiS_2_/MoS_2_/rGO modified GCE in PBS containing 50 μM BPA. Reproduced with permission from [59].

Among TM sulfides, MoS_2_ stands out as the most extensively studied electrode material for the electrochemical sensing of phenol-containing molecules. For instance, MoS_2_ ultrathin nanosheets were synthesized through a one-step pyrolysis process involving ammonium molybdate, thiourea, and layered g-C_3_N_4_ as a sacrificial template. This method prevented restacking of the MoS_2_ nanosheets, resulting in a 3D porous structure. These MoS_2_ nanosheets exhibited enhanced electrocatalytic activity for the simultaneous oxidation of important biomolecules involved in human metabolism, namely ascorbic acid (AA), dopamine (DA), and uric acid (UA). The as-prepared MoS_2_ demonstrated increased peak separation and peak current compared to bulk MoS_2_ (Figure 2a). Moreover, a selective, sensitive, and reproducible simultaneous determination of these biomolecules was successfully achieved using differential pulse voltammetry (DPV). The improved performance was attributed to the exposed Mo-edge sites, high crystallization, and porous structure facilitating electrolyte diffusion [49]. MoS_2_ nanoclusters were also synthetized via a simple hydrothermal treatment without using templates, and their electrochemical behavior for the determination of 4-AP was investigated. The MoS_2_-modified glassy carbon (MoS_2_/GCE) exhibited an LOD of 0.03 µM, enabling the practical determination of 4-AP in environmental samples [50]. Functionalization of MoS_2_ with carbonaceous nanomaterials has also been reported [51,52]. A nanocomposite based on MoS_2_-graphene (MoS_2_-Gr) was prepared and used to construct an electrochemical sensor for acetaminophen detection. The high surface area of graphene and the synergistic effect of MoS_2_ and graphene resulted in better voltammetric response compared to control materials, with a high sensitivity of 3.51 A M^−1^ cm^−2^ [52].

Beyond Mo, other TMs have also been explored for the construction of sensors based on TM sulfides. For instance, nickel disulfide decorated carbon nanotube nanocomposites (N-CNT NCs) were utilized for the detection and measurement of toxic 4-methoxyphenol, while nickel sulfide integrated with mechanical pencil lead (MPL) and reduced graphene oxide (rGO) (MPL-NiS/rGO) was employed for BPA detection [53,54]. Mn, W, and Zn have also been explored to develop TM sulfides for the detection of phenolic compounds, showing promising results [55,56,57].

**Figure 3:**
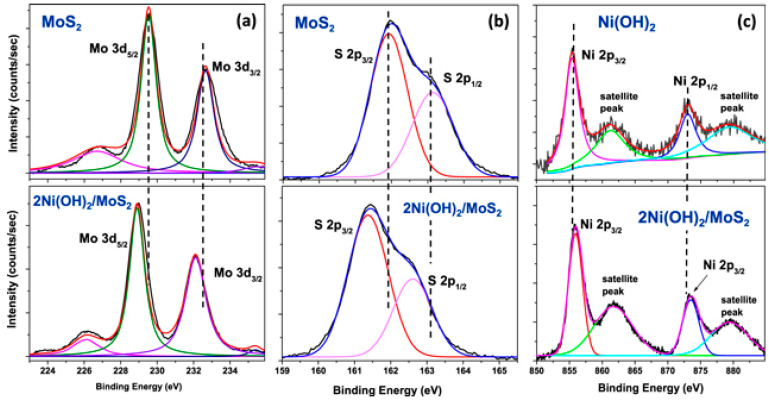
High-resolution XPS spectrum of the following: (**a**) Mo 3d region; (**b**) S 2p region; (**c**) Ni 2p region of MoS_2_, Ni(OH)_2_, and 2Ni(OH)_2_/MoS_2_ composite. Reproduced with permission from [60].

The formation of heterostructures with well-defined heterointerfaces, employing two or more components/phases, is a successful strategy for developing highly active catalysts. The electronic interaction between the different components can modulate the surface/interface properties and redistribute the density of states to optimize the electronic configuration, promoting the adsorption/desorption of reactants/intermediates/products on the surface [58]. Multi-phase nanomaterials, such as NiS_2_/MoS_2_/rGO and Ni(OH)_2_/MoS_2,_ have also been employed for the detection of phenolic molecules, namely BPA and dopamine, respectively. The NiS_2_/MoS_2_/rGO modified GCE exhibited significantly improved oxidation current peaks reaching up to 62.31 μA (Figure 2b) compared to NiS_2_-GCE (10.67 μA) and MoS_2_-GCE (9.63 μA). The incorporation of NiS_2_ microblocks and MoS_2_ nanosheets wrapped by rGO layers increased the number of active sites, providing a relatively larger specific surface area and a more uniform pore-size distribution. This leads to enhanced BPA compared to that of a single component [59]. In the case of Ni(OH)_2_/MoS_2_, different weight ratios between the two phases were prepared using a simple mixing strategy. XPS analysis indicated an electronic interaction between Ni(OH)_2_ and MoS_2_, with shifts in the binding energy values suggesting electron transfer from Ni(OH)_2_ to MoS_2_ (Figure 3). The composition 2Ni(OH)_2_/MoS_2_ (2:1 weight ratio) exhibited the best results in terms of active surface area, R_ct_, LOD, and linear range for dopamine detection [60]. 

#### 2.2.2. Transition Metal Selenides

**Figure 4:**
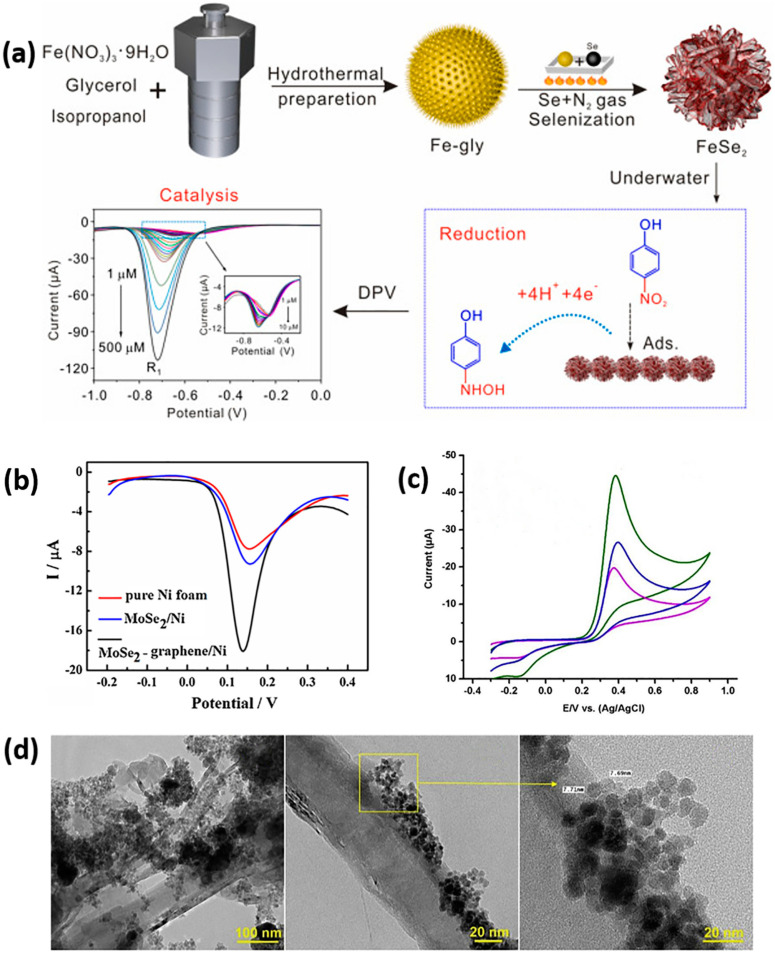
(**a**) Fabrication strategy of FeSe_2_ and detection of 4-NP based on adsorption and catalysis on FeSe_2_ surface. Reproduced with permission from [61]. (**b**) DPVs of different electrodes in 0.1 M PBS (pH 7.0) containing 1 mM dopamine. Reproduced with permission from [65]; (**c**) CV response of H-FeMoSe_2_/SPCE (green curve), M-FeMoSe_2_/SPCE (blue curve), and C-FeMoSe_2_/SPCE (magenta curve) with presence of MES (0.566 mM at 50 mV s^−1^). Reproduced with permission from [71]. (**d**) Different magnified TEM images of CoFeSe_2_/f-CNF nanocomposite. Reproduced with permission from [72].

Transition metal selenides (TMSe) represent a distinct category of inorganic compounds that have been extensively studied in electrocatalysis and energy storage. Cheng et al. prepared FeSe_2_ nanospheres through a hydrothermal synthesis followed by a selenization process. While this material exhibited a diffusion-controlled electron transfer mechanism, it also showed adsorption ability. By combining the effects of adsorption and catalysis, FeSe_2_/GCE was capable of detecting 4-NP and 2-NP (Figure 4a) with a low LOD of 0.030 and 0.034 µM, respectively [61].

Cu_2_Se nanoparticles produced through a one-pot hydrothermal technique have demonstrated high efficiency as an electrochemical sensor for dopamine detection. The Cu_2_Se-modified electrode on carbon cloth facilitated dopamine oxidation at 0.2 V with a high sensitivity of 12.4 A M^−1^ cm^−2^. This sensor performance was attributed to the increased covalency around the catalytically active center, facilitating the catalyst activation step and enhancing charge transfer across the matrix [62]. 

The strategic design and fabrication of self-supported electrodes with a catalytically active phase grown in situ on conductive substrates offer an effective approach to reduce interfacial contact resistance, enhance the physical adhesive force between the catalytic active phase and substrate and eliminate conventional catalyst coating steps [63,64]. In the context of the electrochemical oxidation of dopamine, porous layered molybdenum selenide-graphene (MoSe_2_-graphene) composites were grown in situ on nickel foam. The MoSe_2_-graphene/Ni foam exhibited the largest peak current for dopamine compared to pure Ni foam and MoSe_2_/Ni foam (Figure 4b), with a LOD of 1.0 nM within the linear range of 0.01–10 μM. Moreover, when tested with a 30 nM dopamine solution, the sensor demonstrated good repeatability and reproducibility, presenting a low relative standard deviation (RSD) of 1.6 and 3.8%, respectively [65]. While other TMSe have been explored for dopamine detection, they generally exhibited lower electrochemical performance in terms of sensitivity and LOD [66,67]. Additionally, the formation of TMSe composites with rGO has shown promise for the sensitive detection of 4-NP and 2,4,6-trichlorophenol (TCP). For instance, Ni_3_Se_2_/rGO was electrodeposited on an indium-tin oxide (ITO) electrode, exhibiting fine sensing enhancement toward 4-NP, covering a wide detection range with a low LOD [68]. Furthermore, V_2_Se_9_ nanorods, prepared through a facile hydrothermal process and ultrasonically treated with rGO, demonstrated decent sensitivity for TCP detection. This composite showed good potential for detecting TCP in agricultural soil, water reservoirs, and beverages such as wine and apple juice, owing to the positive synergistic effect between rGO and V_2_Se_9_ and their higher surface area [69]. 

The utilization of TMSe catalysts featuring bimetallic active sites has emerged as a highly effective strategy to improve sensing performance. Notably, the sensing capabilities of these catalysts can be finely tuned by adjusting the molar ratios of the two metals involved. In a study conducted by Ho et al., Co-doped MoSe_2_ at different dopant concentrations (CoMoSe_2_, Co_2_MoSe_2_, Co_3_MoSe_2_, and Co_4_MoSe_2_) and its hybridization with graphene oxide (GO) were investigated for metol sensing [70]. Among the various Co-doped MoSe_2_ ratios, GO@CoMoSe_2_/GCE exhibited a superior electrochemical response, characterized by sharper redox peak current and lower redox peak potential. This enhanced performance was attributed to the maintenance of the layered structural nature of MoSe_2_ at this specific dopant concentration. Comparative studies involving MoSe_2_/GCE, CoMoSe_2_/GCE, and GO/GCE underscore the significant improvement brought about by Co-doping and encapsulation with GO, resulting in a low LOD of 0.009 μM and a sensitivity of 2.397 A M^−1^ cm^−2^. For the detection of mesalazine (MSE) (an anti-inflammatory agent that may cause adverse drug reactions such as pancreatitis and chronic hepatitis in overdosage), a sensor based on a screen-printed carbon electrode (SPCE) and Fe-doped MoSe_2_ was synthesized through hydrothermal, microwave, and chemical methods [71]. The hydrothermal FeMoSe_2_ (H-FeMoSe_2_) variant exhibited a 0.46 and 1.28-fold higher sensing performance for MES compared to other methods (Figure 4c). This superior performance was attributed to H-FeMoSe_2_/SPCE displaying the lowest charge transfer resistance and a favorable morphology that facilitated the exposure of abundant active edge sites. Additionally, H-FeMoSe_2_/SPCE demonstrated good selectivity, with less than 5% current changes in the presence of interferents and satisfactory reproducibility, presenting a relative standard deviation (RSD) of 4.1%. In another instance, CoFeSe_2_ nanospheres anchored on functionalized carbon nanofibers (CNFs) (Figure 4d) were prepared via hydrothermal synthesis for efficient electrochemical oxidation of caffeic acid (CA), a well-known phenolic acid found in various fruits, vegetables, teas, and wines [72]. The composite showcased a low detection limit of 2.0 nM, and the practicality of the CoFeSe_2_/f-CNF/GCE sensor was successfully demonstrated for the detection of CA in real red wine samples without any pre-treatment [72]. Yin et al. embedded cobalt-iron selenide in porous carbon nanofibers to construct a CoFe_2_Se_4_/PCF/GCE sensor [13]. The three-dimensional network structure of PCF facilitated the electron transfer and prevented the aggregation of CoFe_2_Se_4_, resulting in improved electrochemical properties. This sensor demonstrated enhanced performance for the simultaneous sensing of HQ, CC, and resorcinol (RS) [13], which are challenging to detect simultaneously owing to their similar stereochemical structure. Nanoparticles of MoCuSe decorated on rGO via a hydrothermal method proved to be a promising electrode material for the electrochemical sensing of BPA [73]. The sensor exhibited an impressively low LOD of 0.9 nM. When applied to real samples such as milk and drinking water, the sensor presented satisfactory results with recoveries in the range of 98.7% to 102.7%. The authors attribute the good sensor performance to the high surface area and superior electrical conductivity, promoting the accumulation of BPA and enhancing sensitivity toward BPA [73]. In recent developments, a multi-metal selenide comprising of tungsten-doped cobalt-nickel selenide nanosheets arrays (W-Co_0.5_Ni_0.5_Se_2_ NSAs), self-supported on nickel foam served as an effective and accurate sensor for gallic acid detection [74]. Gallic acid is essential for the food and pharmaceutical industry and health perspectives. The W doping into Co_0.5_Ni_0.5_Se_2_ played a significant role in the valence composition of the active sites of Co and Ni, promoting the formation of Co^3+^/Co^2+^ and Ni^3+^/Ni^2+^ redox pairs. This doping enhanced their redox ability and overall electrochemical performance.

#### 2.2.3. Transition Metal Tellurides

**Figure 5:**
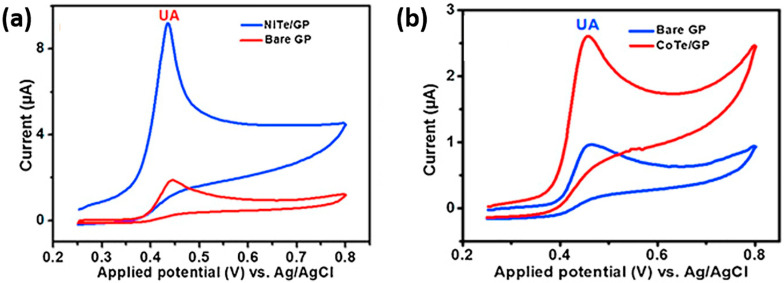
CV curves of the following: (**a**) 0.1 mM UA in 0.1 M phosphate buffer pH 6.0 at bare GP (red) and NiTe/GP (blue) electrodes; (**b**) 0.05 mM UA of at bare GP (blue) and CoTe/GP (red) electrodes. Reproduced with permission from [76].

While the exploration of transition metal tellurides (TMT) for the electrochemical sensing of phenolic compounds is comparatively limited, some noteworthy findings have illuminated their electrochemical activity in detecting phenolic molecules. For instance, the FeTe_2_ nanoparticle-modified graphite paste electrode (FeTe_2_/GP) was investigated for its electrocatalytic oxidation capabilities toward DA and UA biomolecules [75]. The modification of the GP electrode with FeTe_2_ created favorable conditions for efficient electron transfer between the biomolecules and the electrode surface, resulting in proper electrochemical characteristics for their detection. Notably, the FeTe_2_/GP-modified electrode exhibited high sensitivity (reaching 7.29 A M^−1^ cm^−2^ for DA and 6.36 A M^−1^ cm^−2^ for UA) and a wide linear detection range. The successful application of the FeTe_2_ sensor in clinical analysis further highlighted its potential utility [75]. In another study, nanocrystallites of CoTe and NiTe were also modified with a graphite paste (GP) and applied for the detection of UA [76]. Both electrodes demonstrated electrochemical responses to the oxidation of UA. However, NiTe presented a significantly higher oxidation current than CoTe (Figure 5a,b), suggesting enhanced sensitivity for the NiTe/GP sensor, possibly attributed to the superior electrical conductivity of this material. The electrochemical activity of CoTe nanoparticles with thin layered nanosheets and Pt-doped CoTe nanoflakes revealed intriguing findings in dopamine sensing [77]. The study observed that increasing the concentration of Pt in the metal telluride to 5% led to a substantial potential peak shift to lower values accompanied by an increase in the current response. This unique synergy between both metals enhanced the electron density for DA oxidation. The distinctive porous microstructure of 5%Pt-CoTe contributed to achieving a high sensitivity of 24.2 A M^−1^ and an LOD of 24 nM. Moreover, the sensor demonstrated good reproducibility and selectivity.

For the detection of catechol, a nanohybrid incorporating ZnTe nanorods and Au nanoparticles into a copper metal-organic framework was developed for layer-by-layer modification of a GCE. This Cu-MOF/ZnTe NR and AuNP composite exhibited enhanced catalytic properties, benefitting from the synergistic effect of Cu-MOF, ZnTe NRs, and AuNPs. The electrode, under optimized conditions, demonstrated improved sensitivity and selectivity for catechol detection [78]. Another noteworthy application involved a NiTe_2_ nanocrystalline material-modified carbon paste electrode (NiTe_2_/CPE), resulting in a sensitive and selective sensor for voltammetric determination of the antioxidant molecule morin in red wines. This sensor displayed high sensibility and a wide linear range suitable for real-time sample analysis within a complex matrix [79]. The versatility of TMT was further demonstrated with Ni_3−x_Te_2_ for the simultaneous detection of the neurotransmitters DA and adrenaline (AD) [80] and CoTe_2_ nanocrystals for determining ferulic acid in cosmetic analysis [81], yielding satisfactory results.

### 2.3. Transition Metal Phosphides

Amidst emerging catalysts, transition metal phosphides (TMPs) have recently garnered attention as promising electrode materials for molecular detection. Despite the significance of phenolic substances as environmental contaminants, only a few works have reported the use of monometallic TMPs for their detection [82,83,84,85].

**Figure 6:**
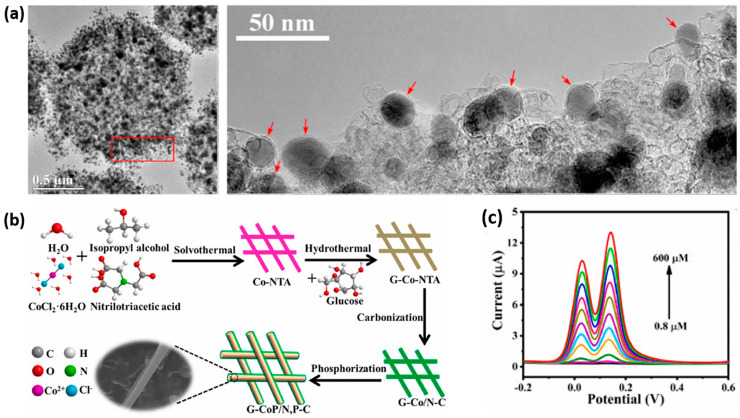
(**a**) TEM images of Co_x_P/NC and high-magnification TEM images taken from the red square. The red arrows show cobalt phosphide nanoparticles encapsulated with some graphene layers. Reproduced with permission from [82]. (**b**) Schematic illustration of the formation of G-CoP/N,P–C. (**c**) DPVs of G-CoP/N,P–C/GCE in 0.1 M PBS (pH 8.0) with various concentrations of HQ and CC. Reproduced with permission from [85].

The metal-organic framework (MOF) has become a versatile platform for fabricating nanostructured electrocatalysts, leveraging its substantial specific surface area, spatially-ordered microstructure, and high porosity. Through the utilization of MOF-derived nanomaterials, a cobalt phosphide embedded within nitrogen-doped porous carbon microspheres (Co_x_P/NC, Figure 6a) was prepared using one-step phosphorization and carbonization for the electrochemical oxidation of 4-NP. The Co_x_P@NC exhibited a large surface area (826 m^2^ g ^−1^) and abundant mesopores. When used to modify a GCE, a 6-fold enhancement in current was obtained compared to NC/GCE [82], leading to a low LOD of 2 nM. Similarly, MOF-derived CoP_x_ polyhedrons demonstrated excellent electrochemical performance for 4-NP detection, with a LOD of 0.79 nM and a high sensitivity of 802 A M^−1^ cm^−2^ attributed to their superior electrical conductivity and high active surface area [84]. For the simultaneous determination of HQ and CC, nitrogen and phosphorous co-doped glucose-derived carbon-coated CoP nanowires (G-CoP/N,P–C NWs) were developed using multi-step reactions (Figure 6b). By DPV, two well-separated oxidation peaks were observed (Figure 6c), and the corresponding electrochemical oxidation currents of the two isomers increased linearly with concentration over a wide concentration range [85]. Other monometallic TMPs, such as Ni_2_P nanosheets [86] and nitrogen and iron phosphides doped carbon nanotubes (N/FeP-CNT) [83], have also demonstrated efficiency as electrode materials for the electrochemical detection of acetaminophen and simultaneous detection of dihydroxybenzoic acid isomers, respectively.

**Figure 7:**
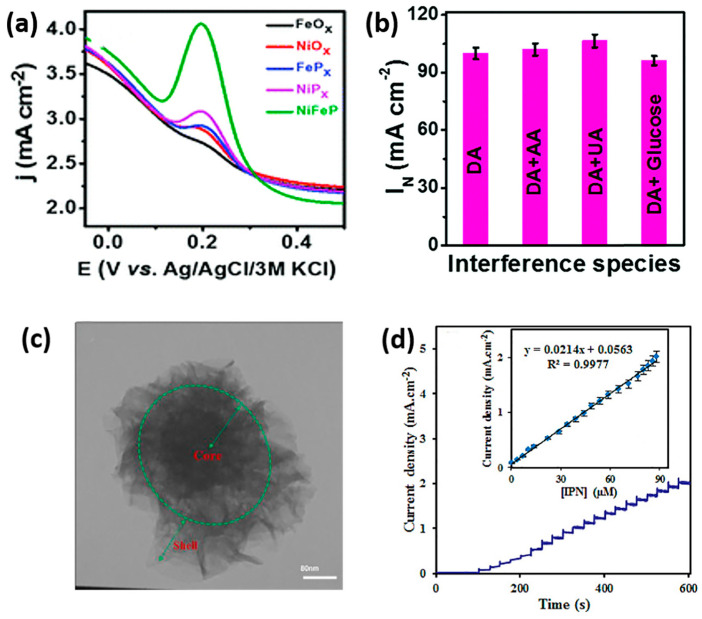
(**a**) SWV results for various catalysts in 0.1 M PBS electrolyte containing 50 µM DA and 200 µM AA. (**b**) Bar diagram of the normalized current in the presence of interferents. Reproduced with permission from [90]. (**c**) TEM image of as-prepared NiCo-P@NiCo-LDH. (**d**) Amperogram of the NiCo-P@NiCo-LDH/GCE at various IPN concentrations. Inset is the related calibration curve. Reproduced with permission from [95].

Recently, bimetallic TMPs have also been explored for the detection of phenolic pollutants. For example, a nanocomposite of MnCo-P onto sulfur-doped reduced graphene oxide (S-RGO) (MnCo-P/S-RGO) was developed for the detection of acetaminophen. MnCo-P exhibited increased redox response in both current and peak-to-peak separation compared to MnCo-layered double hydroxide (LDH). The synergistic activity between MnCo-P and S-RGO significantly improved the catalytic performance, achieving a sensitivity of 0.658 A M^−1^ cm^−2^. The sensor exhibited good repeatability and reproducibility, boasting low relative standard deviation (RSD) values of 1.09% and 0.14%, respectively [87]. Another study delved into a heterojunction composed of CoP-NiCoP nanosheets supported on a graphene framework (CoP-NiCoP/GFs). This heterojunction exhibited enhanced electrocatalytic activity toward HQ and CC compared to CoP/GFs, NiCoP/GFs, and GFs alone. The formation of a heterojunction interface optimized the chemisorption of HQ and CC, reducing the activation energy for the hydrogen dissociation reaction and facilitating the electrocatalytic oxidation reaction of both molecules [88]. This class of catalysts has also demonstrated sensitive detection capabilities for various molecules, including dopamine [89,90,91,92,93], isoprenaline [94,95], and chloramphenicol [96]. For instance, a self-supported NiCoP into Ni foam was synthesized using one-step electrodeposition for DA oxidation. The morphologies evolved from nanoparticles to spheres within 20 min, and the best electrochemical activity was achieved for NiCoP electrodeposited for 10 min, exhibiting vertically standing nanosheets on the surface of Ni foam along with dispersive spheres cluster. Due to the highest surface area, NiCoP exhibited a LOD of 1 µM and a sensitivity of 5.26 A M^−1^ cm^−2^ [92]. Similarly, an electrochemical microfluidic sensor based on graphene fiber microelectrodes modified using hierarchically porous NiCoP nanosheets demonstrated comparable sensitivity (5.56 A M^−1^ cm^−2^) and improved LOD (14 nM) for DA sensing [93]. In a comprehensive study, Thakur et al. investigated nickel-iron phosphide/phosphate (NiFeP) nanosheets as an efficient catalyst for the selective and sensitive determination of DA. The NiFeP/GCE sensor exhibited an improved electrochemical response toward DA oxidation compared to monometallic NiP and FeP catalysts, outperforming their respective oxides (Figure 7a). Additionally, the use of NiFeP as a flexible electrode, coated onto Whatman filter paper, displayed an impressive sensibility of 756 A M^−1^ cm^−2^, even in the presence of interferents such as ascorbic acid (AA) (Figure 7b) [90].

The hybridization of the active catalysts with other components has emerged as a strategic approach for elevating catalytic performance. A noteworthy example is the development of a hybrid core-shell nanostructure, NiCo-P@NiCo-LDH, designated for the detection of isoprenaline, an extensively used drug in medical treatment such as allergic emergencies and hypertension. The transmission electron microscopy (TEM) analysis unveiled a uniform coating of NiCo-LDH nanosheets on the surface of NiCo-P nano-discs (Figure 7c). Extensive characterization revealed the hybrid’s unique features, including a large surface area, high conductivity, and a synergistic effect among Ni and Co ions in the core. This configuration resulted in a high-performance sensing platform for the determination of isoprenaline (Figure 7d) [95]. Amorim et al. investigated the role of different components, such as P and rGO, on the catalytic performance of CoNiP nanoparticles anchored on rGO (CoNiP@rGO) for HQ detection. Their findings underscored the synergistic interaction between CoNiP and rGO, where P increases the proton concentration at the electrode interface, favoring a catalytic mechanism involving the oxidation of metal centers. The presence of rGO suppressed this effect due to the formation of high valence states of CoNiP, leading to a markedly improved electrochemical response (approximately with ten times higher current densities and lower peak-to-peak separation) compared to rGO and samples in the absence of P and/or rGO [97]. Building upon these insights, the same authors selected the CoNiP@rGO composite as the primary material for further studies, specifically evaluating the performance parameters for BPA detection. The results indicated that the detection process follows an adsorption-controlled electron transfer mechanism, enhancing electrocatalytic activity for BPA at concentrations below the regulatory limits [98].

A comparative overview of the sensing performance parameters of phenolic compounds for the different classes of materials is synthetized in Table 1.

**Table 1:** Comparative performance of different electrode materials for phenolic compound detection.

Electrode Material	Phenolic Compound	Method	Linear Range (µM)	Sensitivity (A M^−1^ cm^−2^)	LOD (µM)	Ref.
O–Cu_2_O/GCE	4-aminophenol	DPV	0.008–9	-	0.0018	[39]
	4-chlorophenol		0.01–4	-	0.0027	
	4-nitrophenol		0.08–30	-	0.0085	
Co_3_O_4_/GCE	Uric acid	DPV	0–1500	2.16	1.6	[40]
Co_3_O_4_/GCE	Hydroquinone	DPV	1–500	0.721	0.1	[41]
	Catechol		1–500	0.354	0.1	
CuCo_2_O_4_/GCE	Metol	DPV	0.02–1000	1.45	0.006	[43]
CoFe_2_O_4_/GCE	Bisphenol A	DPV	0.05–10	0.815	0.0036	[44]
ZnFe_2_O_4_/g-C_3_N_4_/GCE	4-nitrophenol	Amp.	0.015–724	1.68	0.0042	[45]
ZnFe_2_O_4_/PANI@rGO/GCE	4-nitrophenol	DPV	1–100	36.9	0.083	[46]
MWCNTs/CuFe_2_O_4_/GCE	Bisphenol A	DPV	0.01–120	5.07	0.0032	[47]
MoS_2_/GCE	Ascorbic acid	DPV	5–1200	0.16	0.82	[49]
	Dopamine		1–900	0.72	0.15	
	Uric acid		1–60	10.13	0.06	
MoS_2_/GCE	4-aminophenol	DPV	0.04–7	0.0043	0.03	[50]
GNS-CNTs/MoS_2_/GCE	Dopamine	DPV	0.1–100	10.81	0.05	[51]
MoS_2_-Gr/GCE	Acetaminophen	DPV	0.1–100	3.51	0.02	[52]
NiS_2_/MoS_2_/rGO/GCE	Bisphenol A	DPV	0.02–200	0.2646 A M^−1^	0.0021	[59]
Ni(OH)_2_/MoS_2_/GCE	Dopamine	DPV	0.75–95	0.0284 A M^−1^	0.056	[60]
MPL-NiS/rGO	Bisphenol A	ASV	0.043–0.26	-	1.75	[54]
MnS/GCE	Bisphenol A	DPV	0.02–109	-	0.0065	[55]
WS_2_-Gr/GCE	Catechol	DPV	1–100	0.447	0.2	[57]
	Resorcinol			0.206	0.1	
	Hydroquinone			0.380	0.1	
FeSe_2_/GCE	4-nitrophenol	DPV	1–10	0.397 A M^−1^	0.030	[61]
	2-nitrophenol		1–10	0.377 A M^−1^	0.034	
Cu_2_Se/CC	Dopamine	Amp.	0.002–30	12.4	0.084	[62]
MoSe_2_-graphene/Ni foam	Dopamine	DPV	0.01–10	0.104 A M^−1^	0.001	[65]
Pt/Co_0.85_Se/GCE	Dopamine	DPV	0.5–22	2.31	0.39	[66]
S-MoSe_2_/NSG/Au/MIPs/GCE	Dopamine	DPV	0.05–100	0.101 A M^−1^	0.02	[67]
Ni_3_Se_2_/rGO/ITO	4-nitrophenol	DPV	0.05–5	3.06 A M^−1^	0.017	[68]
V_2_Se_9_/rGO/GCE	2,4,6-trichlorophenol	DPV	0.001– 1150	0.0184 A M^−1^	0.035	[69]
GO@CoMoSe_2_/GCE	Metol	DPV	0.04–40	2.39	0.009	[70]
H-FeMoSe_2_/SPCE	Mesalazine	DPV	0.004–57	0.24	0.008	[71]
CoFe_2_Se_4_/PCF/GCE	Hydroquinone	DPV	0.5–200	0.814	0.13	[13]
	Catechol		0.5–190	0.829	0.15	
	Resorcinol		5–350	0.357	1.36	
CoFeSe_2_/f-CNF/GCE	Caffeic acid	DPV	0.01–264	2.04	0.002	[72]
MoCuSe-rGO/GCE	Bisphenol A	DPV	0.003–0.9	12.86	0.0009	[73]
W-Co_0.5_Ni_0.5_Se_2_/Ni foam	Gallic acid	DPV	1–36.2	1.33 A M^−1^	0.120	[74]
FeTe_2_/GP	Dopamine	DPV	5–120	7.29	0.028	[75]
	Uric acid		3–120	6.36	0.042	
NiTe/GP	Uric acid	DPV	3–200	0.108 A M^−1^	0.095	[76]
5%Pt-doped CoTe/GCE	Dopamine	DPV	0.049–0.84	342.2	0.025	[77]
Cu MOF/ZnTe/Au/GCE	Catechol	DPV	0.25–300	0.142 A M^−1^	0.016	[78]
NiTe_2_/CPE	Morin	DPV	0.014–32	5.42 A M^−1^	0.0133	[79]
CPE/Ni_3-x_Te_2_	Dopamine	SWV	4–31	1.12 A M^−1^	0.15	[80]
	Adrenaline		4–31	0.64 A M^−1^	0.35	
CoTe_2_/GCE	Ferulic Acid	SWV	0.04–28	1.096 A M^−1^	0.013	[81]
Co_x_P/NC/GCE	4-nitrophenol	DPV	0.05–1	20.9 A M^−1^	0.002	[82]
CoP_x_@NCNTs/GCE	4-nitrophenol	LSV	0.0025–1	802	0.00079	[84]
G-CoP/N,P–C/GCE	Hydroquinone	DPV	0.8–900	0.541	0.18	[85]
	Catechol	DPV	0.6–800	0.986	0.12	
Ni_2_P/GCE	Acetaminophen	Amp.	0.5–4500	0.131	0.107	[86]
MnCo-P/S-RGO/RRDE	Acetaminophen	Amp.	0.05–1.94	0.658	0.00139	[87]
CoP-NiCoP/GFs/GCE	Hydroquinone	DPV	1–101	0.18 A M^−1^	0.256	[88]
	Catechol		2–102	0.21 A M^−1^	0.379	
CC/Ti_3_C_2_Tx/NiCoP	Dopamine	Amp.	0.17–785	31.4	0.00018	[89]
NiFeP/Whatman filter paper	Dopamine	SWV	0.01–10	756	0.0001	[90]
CoP/Ti mesh	Dopamine	Amp.	1–3000	3.36	0.356	[91]
NiCoP/Ni foam	Dopamine	Amp.	0.5–2350	5.26	1	[92]
NiCoP/GF microelectrode	Dopamine	DPV	0.5–200	5.56	0.014	[93]
ZnNiP/GCE	isoprenaline	Amp.	0.2–5000	0.0668 A M^−1^	0.06	[94]
NiCo-P@NiCo-LDH/GCE	isoprenaline	Amp.	0.5–2110	21.4	0.17	[95]
CoP_x_-N-C/GCE	chloramphenicol	DPV	0.2–40	0.181 A M^−1^	0.044	[96]
CoNiP@rGO/GCE	Hydroquinone	DPV	0.5–25	36.4	0.5	[98]
	Bisphenol A		0.001–8	96.4	0.38	

DPV—Differential Pulse Voltammetry; Amp.—Amperometry; ASV—Anodic Stripping Voltammetry; SWV—Square Wave Voltammetry; LSV—Linear Sweep Voltammetry.

## 3. Conclusions and Future Perspectives

This comprehensive review provides insights into the recent advancements in various families of transition metal-based electrocatalysts employed in the fabrication of electrochemical sensors for phenolic compounds—a significant endeavor with applications spanning healthcare, water quality, and food safety. These materials exhibit exceptional performance in producing electrochemical sensors with distinctive responses, showcasing remarkable sensitivity across different phenolic molecules and applications, thereby presenting extensive opportunities in the sensing domain. The escalating research interest in transition metal-based electrocatalysts is attributed to their unique physical and chemical properties, positioning them as promising electrode materials across diverse applications. Moreover, the use of transition-metal-based materials allows the development of electrode materials that are highly sensitive to phenolic substances using materials that are abundant and of lower prices in comparison to the traditional electrode materials. 

While monometallic TMs have undergone extensive exploration, the emergent trend toward multimetallic TMs is noteworthy. Such materials manifest a synergistic effect resulting from the combination of different transition metal species, amplifying the number of electrochemically active sites and enabling facile modulation of the electronic structure—a key aspect contributing to favorable electrochemical properties. Furthermore, hybrid nanostructures offer opportunities for customized surface functionalization, facilitating specific interactions with phenolic compounds that can enhance the selectivity of the sensor. The integration of carbon-based nanostructures has proven significantly beneficial in augmenting the efficiency and effectiveness of TM-based electrocatalysts.

An analysis of Table 1 reveals that, in general, transition metal phosphides emerge as the leading family of materials concerning LOD and/or sensitivities for various phenolic compounds, such as dopamine, hydroquinone, and 4-nitrophenol. Transition metal selenides closely follow, indicating a promising avenue for the detection of non-explored phenolic compounds within these TM families. In the pursuit of identifying the most appropriate transition metal (TM) for detecting specific phenolic compounds, an examination of Table 1 brings to light discernible trends. Notably, Ni-based materials prove exceptionally well-suited for dopamine detection, while Co-based materials emerge as notably relevant in detecting 4-nitrophenol. Additionally, Cu-based materials demonstrate the most favorable results for detecting BPA. Readers are encouraged to acknowledge that the selection of a suitable transition metal material for the detection of a specific phenolic compound is contingent upon various factors. These factors include the electrocatalytic properties of the materials, encompassing morphology, structure, size, conductivity, and stability. Furthermore, the chosen detection method assumes a pivotal role and can significantly influence the limits of detection (LODs) and sensitivities. Consequently, determining the most reliable transition metal is not a straightforward task. In this context, the application of density functional theory (DFT) calculations becomes instrumental. DFT calculations wield significant influence and offer valuable insights in studies aimed at facilitating the selection of the optimal TM and confirming its efficacy in sensor applications.

This review also aims to address the challenges inherent in the detection of phenolic compounds. A notable challenge arises from the structural analogs of phenolic molecules, which frequently share similar oxidation potentials, thereby complicating simultaneous detection using electrochemical sensors. The comprehensive analysis presented in this review highlights the pivotal role that transition metal-based materials can play in overcoming this challenge. These materials exhibit enhanced performance in simultaneously sensing structurally similar compounds, such as HQ, CC, and resorcinol. Furthermore, many of the transition metal-based electrocatalysts discussed in this review are nanostructures. Nanostructures are recognized for providing a large redox-active surface area, facilitating the overcoming of high redox potentials associated with phenolic compounds. This characteristic results in heightened oxidation currents even at low analyte concentrations, thereby contributing to improved sensitivity and responsiveness of the sensor. Moreover, certain phenolic compounds (e.g., uric acid and dopamine) are only electrochemical detectable by using biomolecules at the electrode surface (e.g., enzyme-based sensors), which can present challenges due to intricate immobilization processes, elevated costs, and limited repeatability. In this context, transition metal-based catalysts emerge as a viable alternative for the biomolecule-free detection of these molecules. 

Despite substantial advancements in catalyst design and synthesis, continued efforts are imperative to enhance electrocatalytic performance further. The integration of theoretical predictions with advanced in situ and operando spectroscopic and microscopic characterization techniques holds promise for providing fundamental insights into active species and catalytic/degradation mechanisms. This integrated approach will facilitate the rational design of catalysts, thereby significantly improving electrocatalytic performance. 

Furthermore, to broaden the analytical scope of the TM-based electrochemical sensors, future research should focus on the utilization of real samples such as industrial wastewater, human urine, and various commercial products, including hair dyes, skin creams, packaged food, and beverages. Aligning the sensitivities and detection limits achieved by these electrocatalysts in relevant matrices with safety standards set by regulatory and monitoring agencies is essential for ensuring practicality and relevance.

The mass production of sensors for personal use is envisaged for the near future, thanks to the advancement of screen printing and thin-layer technologies, among others. The adoption of these devices in diverse applications and across developing countries can be expedited by employing transition metal-based materials. These materials, owing to their relatively high availability in the Earth’s crust, have the potential to reduce production costs, making electrochemical sensors more accessible for mass production. In addition, establishing more straightforward methodologies and accessible alternatives for the preparation and deployment of these sensors is essential to enhance accessibility and democratize the availability of advanced analytical techniques, particularly in regions with limited resources.

In summary, the insights provided in this review offer a pathway for the development of diverse combinations of transition metals, paving the way for advanced and novel electrocatalysts capable of detecting phenolic molecules at trace levels.

## Data Availability

Data are contained within the article.
